# A large-scale assessment of sequence database search tools for homology-based protein function prediction

**DOI:** 10.1093/bib/bbae349

**Published:** 2024-07-22

**Authors:** Chengxin Zhang, Lydia Freddolino

**Affiliations:** Department of Computational Medicine and Bioinformatics, Department of Biological Chemistry, University of Michigan, 100 Washtenaw Avenue, Ann Arbor, MI 48109, United States; Department of Computational Medicine and Bioinformatics, Department of Biological Chemistry, University of Michigan, 100 Washtenaw Avenue, Ann Arbor, MI 48109, United States

**Keywords:** Gene Ontology, protein function prediction, sequence database search, BLASTp, DIAMOND, MMseqs2

## Abstract

Sequence database searches followed by homology-based function transfer form one of the oldest and most popular approaches for predicting protein functions, such as Gene Ontology (GO) terms. These searches are also a critical component in most state-of-the-art machine learning and deep learning-based protein function predictors. Although sequence search tools are the basis of homology-based protein function prediction, previous studies have scarcely explored how to select the optimal sequence search tools and configure their parameters to achieve the best function prediction. In this paper, we evaluate the effect of using different options from among popular search tools, as well as the impacts of search parameters, on protein function prediction. When predicting GO terms on a large benchmark dataset, we found that BLASTp and MMseqs2 consistently exceed the performance of other tools, including DIAMOND—one of the most popular tools for function prediction—under default search parameters. However, with the correct parameter settings, DIAMOND can perform comparably to BLASTp and MMseqs2 in function prediction. Additionally, we developed a new scoring function to derive GO prediction from homologous hits that consistently outperform previously proposed scoring functions. These findings enable the improvement of almost all protein function prediction algorithms with a few easily implementable changes in their sequence homolog-based component. This study emphasizes the critical role of search parameter settings in homology-based function transfer and should have an important contribution to the development of future protein function prediction algorithms.

## Introduction

Homology-based function transfer is a classical and widely used approach for predicting protein functions. In this method, the sequence of the query protein is searched through a database of template proteins with function annotations. The biological functions of the query protein are then derived from the annotations of the top sequence search hits. The operation of searching for sequence homologs forms the foundation for many classical protein function predictors [1–10] and remains a critical component for numerous state-of-the-art deep learning-based function prediction algorithms [11–16] (Table 1).

**Table 1:** Usage of sequence search tools and machine learning algorithms by different protein function prediction programs

Function prediction program	Machine learning	Year introduced	Sequence search tool	Scoring function
GOtcha [1]	None	2004	BLASTp	Other
Blast2GO [2]	None	2008	BLASTp	Other
ConFunc [3]	None	2008	PSI-BLAST	Other
PFP [4]	None	2009	PSI-BLAST	Other
BAR-PLUS [5]	None	2011	BLASTp	Other
CombFunc [6]	SVM	2012	PSI-BLAST, BLASTp	Other
GoFDR [7]	None	2016	PSI-BLAST	Other
COFACTOR [8]	None	2017	PSI-BLAST, BLASTp	*S*_3_
MetaGO [9]	Logistic regression	2018	PSI-BLAST, BLASTp	S_3_
GOLabeler [17]	Logistic regression, gradient boosted tree	2018	BLASTp	*S*_1_
HFSP [10]	None	2018	MMseqs2	*S*_10_
NetGO [18]	Logistic regression, gradient boosted tree	2019	BLASTp	*S*_1_
DeepGOplus [11]	Deep learning	2020	DIAMOND	*S*_1_
TALE [12]	Deep learning	2021	DIAMOND	*S*_1_
ATGO [13]	Deep learning	2022	BLASTp	*S*_1_
DeepGOZero [14]	Deep learning	2022	DIAMOND	*S*_1_
ProtInfer [15]	Deep learning	2023	BLASTp	*S*_1_
SPROF-GO [16]	Deep learning	2023	DIAMOND	Other

Despite the central role of sequence database search tools in homology-based function prediction, there is no consensus on the best tool and its optimal search parameters for predicting biological functions. Until the year 2018, most function predictors, which typically used no machine learning or only traditional (non-deep) machine learning, derived protein functions from hits found by BLASTp or PSI-BLAST [1–9, 17]. BLASTp accelerates sequence-sequence alignment by prefiltering sequence database entries using exact matches of short fragments (referred to as k-mers) before performing dynamic programming-based sequence alignment [19]. PSI-BLAST extends BLASTp into iterative profile-sequence alignment, where the query sequence profile is represented by a position specific scoring matrix constructed from multiple sequence alignment (MSA) from the last iteration [19]. On the other hand, from 2020 onwards, DIAMOND [20] began to replace (PSI-)BLAST in many function predictors [11, 12, 14, 16]. DIAMOND is meant to provide a BLASTp alternative optimized for speed [20]. This is partly achieved by a reduction of amino acid alphabet from 20 standard amino acid types to only 11 to make the k-mer prefiltering more efficient at the expense of a small loss in sensitivity. Similar approaches have been implemented by other sequence search tools such as RAPsearch2 [21], but DIAMOND is the first tool that can achieve sensitivity comparable to (albeit still slightly worse than) that of BLAST. We note that while the introduction of DIAMOND into protein function prediction pipelines coincided with the wide adoption of deep learning for function prediction, there is no direct relation between DIAMOND and deep learning. Although DIAMOND can achieve a 100-fold speed up compared with BLASTp, its sensitivity is still lower than BLASTp. This is part of the reason that some recent predictors still prefer BLASTp over DIAMOND [13, 15]. An even faster sequence search tool is MMseqs2 [22], which further improves the efficiency of the prefiltering stage by allowing inexact k-mer matches followed by vectorized dynamic programming alignment. Although MMseqs2 runs at a speed that is at least comparable if not faster than DIAMOND, it is used by only one major function predictor (HFSP) to our knowledge [10]. Another fast sequence search tool is GHOSTX [23], which sees limited usage in function annotations. It implements a fast k-mer prefiltering by indexing k-mers for both query and database sequence. To further reduce computational cost, GHOSTX performs ungapped extension of matched k-mers to make as much ungapped alignment as possible before performing the more expensive gapped alignment.

In parallel to the k-mer matching-based heuristic sequence search tools described above, more sensitive hidden Markov Model (HMM)-based sequence database search tools have also been developed and widely applied to protein structure prediction [24–26]. Two popular packages for HMM-based sequence search are HMMER [27] and HHblits [28]. The HMMER package provides the phmmer tool, which constructs an HMM from the query protein sequence using a simple position-independent scoring (BLOSUM62 matrix scores converted to probabilities, plus the probabilities for gap openings and gap extensions). This HMM is matched to database sequences to identify homologs. HMMER also provides jackhammer, which is an iterative version of phmmer where the query HMM is updated in each iteration based on the alignment of identified homologous hits. While both phmmer and jackhmmer directly search sequence databases, HHblits searches HMM databases. To this end, performs HMM-HMM alignment on preclustered sequence database. Specifically, HHblits first cluster the sequence database and perform MSA within each cluster. Each MSA is then converted to an HMM model. The profile columns in the HMM are further discretized into an alphabet of 219 states, where each letter represents a typical profile column. This allows an approximation of an HMM by a 219-letter sequence. During the search of a query through the sequence database, the query is also converted to an HMM and a 219-letter sequence, which is then used to prefilter the HHblits database to drastically reduce the number of alignments necessary. Despite the popularity of HHblits and HMMER in structure prediction, they are rarely used for sequence database search during homology-based function prediction, although HMMER is indeed used for the search of protein families for some function prediction pipelines [17, 18]. The multitude of available tools for sequence database searches calls for systematic comparisons in order to identify optimal approaches for protein function prediction, which will not necessarily coincide with those for structure prediction. More recently, machine learning tools [29, 30] have been developed to perform protein sequence search and alignment based on the comparison of embeddings of protein sequence generated by protein language models. While these tools, such as PLMSearch [29], show promise in identifying related sequences with similar structures, they were not developed or evaluated for function predictions.

Beyond the choice of sequence database search tool, the scoring function used to derive function annotations from a specific set of template proteins, i.e. the set of homologous hits identified by the sequence search tool, also plays a major role in the accuracy of homology-based function prediction. For example, several recent studies [9, 17] consistently indicate that deriving the prediction score from multiple hits tends to produce more accurate predictions than simply deriving the function prediction from the template with the highest sequence identity. However, whether different sequence search tools should employ different scoring functions remains an open question.

To tackle these questions, we performed a direct comparison of popular sequence search tools used for protein structure and function prediction, as well as a set of scoring functions for homology-based function prediction. Working in the context of a large-scale protein Gene Ontology (GO) prediction task, we observed a profound effect of the choices of search tool, tool-specific parameters, and scoring function on the overall performance. Our findings provide a workflow for homology-based function prediction that appears optimal both in terms of accuracy and speed on the datasets used in our comparisons and provide a strong foundation for ongoing efforts in tool development for protein function prediction.

## Materials and methods

### Protein sequence database search tools

We evaluated seven popular protein sequence database search tools: BLASTp [19], DIAMOND [20], MMseqs2 [22], PSI-BLAST [19], phmmer [27], jackhmmer [27], and HHblits [28]. Among these, BLASTp (version 2.13.0+), DIAMOND (version 2.1.8.162), and MMseqs2 (version 390457d87ed7049d918e46bc8b0571ac4034aae4) are based on sequence-sequence alignment. PSI-BLAST (version 2.13.0+) by default perform sequence–sequence alignment, producing results identical to those from BLASTp. Rather than using the default setting, we utilized the ‘-num_iterations 3’ option to perform an iterative profile-sequence search with PSI-BLAST. These four programs can utilize a protein sequence database after a straightforward reformatting process to convert the database text into binary formats.

Jackhmmer (version 3.4) and phmmer (version 3.4) are based on HMM-sequence alignment. Both programs can directly search a FASTA format sequence database.

On the other hand, HHblits (version 2.0.15) is based on iterative HMM-HMM alignment and requires an HHblits-format database of HMMs. To construct such a database, we follow our previous protocol [24]. Briefly, all template proteins with GO annotations are grouped into sequence clusters by kClust [31] using a 30% sequence identity cutoff. We then use Clustal Omega [32] to align sequences within each cluster into aligned sequence profiles. These profiles are fed into the hhblitsdb.pl script accompanying the HHblits software to construct the HHblits-format database.

### Scoring functions for function prediction

We implemented 11 different scoring schemes to derive function predictions from a set of template hits identified by sequence database search. In the first scoring function, the score for predicting GO term *q* is


(1) \begin{equation*} {S}_1(q)=\frac{\sum_{k=1}^{K(q)}{bitscore}_k(q)}{\sum_{k=1}^K{bitscore}_k}. \end{equation*}

Here, ${bitscore}_k$ is the bit-score for the *k*th template; *K* is the total number of templates with GO annotations; ${bitscore}_k(q)$ and *K*(*q*) are the respective values for the subset of templates with GO term *q*. This scoring function, where each template is weighted by bit-score, appears to be the most popular scoring function among recently developed machine learning-based function predictors [11, 13, 17, 18]. It was called either BLAST-KNN [17] or DiamondScore [11] by previous studies.

The second scoring function is newly introduced by this study, where the template is weighted by both the bit-score and sequence identity.


(2) \begin{equation*} {S}_2(q)=\frac{\sum_{k=1}^{K(q)}{bitscore}_k(q)\cdot{ID}_k(q)}{\sum_{k=1}^K{bitscore}_k\cdot{ID}_k}. \end{equation*}

Here, ${ID}_k$ is the sequence identity for the *k*th template, calculated by the number of identical residues divided by the maximum between the query sequence length and template sequence length. ${ID}_k(q)$ is the respective value for the *k*th template with GO term *q*. While both the bit-score and sequence identity quantify the similarity between the query and template, they provide complementary information. Specifically, the bit-score is derived from the gap penalties and scoring matrix (such as BLOSUM62) used to align the query and template; therefore, it is highly correlated with the parameter set up of the sequence search tool. On the other hand, the sequence identity is a more intrinsic property for the query-template sequence pair and is less search-tool-specific. This is why the combination of the two metrics provides more information than using either metric alone, as shown in Results section.

The third scoring function was introduced by MetaGO [9], where the template is weighted by ${qID}_k$, the sequence identity of the *k*th template normalized by the length of query sequence


(3) \begin{equation*} {S}_3(q)=\frac{\sum_{k=1}^{K(q)}{qID}_k(q)}{\sum_{k=1}^K{qID}_k}. \end{equation*}

The fourth, fifth, and sixth scoring function are defined similarly, except that the sequence identities ${tID}_k$, ${aID}_k$, and ${ID}_k$ are normalized by the length of the *k*th template, the number of aligned residues, and the maximum between query and template, respectively


(4) \begin{equation*} {S}_4(q)=\frac{\sum_{k=1}^{K(q)}{tID}_k(q)}{\sum_{k=1}^K{tID}_k} \end{equation*}

(5) \begin{equation*} {S}_5(q)=\frac{\sum_{k=1}^{K(q)}{aID}_k(q)}{\sum_{k=1}^K{aID}_k} \end{equation*}

(6) \begin{equation*} {S}_6(q)=\frac{\sum_{k=1}^{K(q)}{ID}_k(q)}{\sum_{k=1}^K{ID}_k}. \end{equation*}

The seventh scoring function is the frequency of templates with GO term *q* among all templates


(7) \begin{equation*} {S}_7(q)=\frac{K(q)}{K}. \end{equation*}

Whereas the previous scoring functions are all calculated from all template hits, the remaining four scoring functions only consider the template with the highest sequence identity among all templates with GO term *q*. In the 8th, 9th, 10th, and 11th scoring functions, the sequence identities are normalized by the query length, template length, number of aligned residues, and the maximum between query length and template length, respectively.


(8) \begin{equation*} {S}_8(q)=\underset{k}{\max}\left(\ {qID}_k(q)\ \right) \end{equation*}

(9) \begin{equation*} {S}_9(q)=\underset{k}{\max}\left(\ {tID}_k(q)\ \right) \end{equation*}

(10) \begin{equation*} {S}_{10}(q)=\underset{k}{\max}\left(\ {aID}_k(q)\ \right) \end{equation*}

(11) \begin{equation*} {S}_{11}(q)=\underset{k}{\max}\left(\ {ID}_k(q)\ \right). \end{equation*}

### Evaluation of function prediction performance

The performance of protein function prediction is usually evaluated by the maximum *F*-measure (Fmax) and/or the maximum of information content-weighted *F*-measure (wFmax). Fmax was the main evaluation metric for Critical Assessment of Function Annotation (CAFA) challenges round 1, 2, and 3 [33], and is defined as


(12) \begin{equation*} F\max =\underset{t\in \left(0,1\right]}{\max}\left(\frac{2\cdot pr(t)\cdot re(t)}{pr(t)+ re(t)}\right). \end{equation*}

Here, *pr*(*t*) and *re*(*t*) are the precision and recall, respectively, at the prediction score threshold *t*. They are defined as


(13) \begin{equation*} pr(t)=\frac{1}{M(t)}\sum_{i=1}^{M(t)}\frac{\sum_qI\left[q\in{P}_i(t)\bigwedge q\in{T}_i\right]}{\sum_qI\left[q\in{P}_i(t)\right]} \end{equation*}

(14) \begin{equation*} re(t)=\frac{1}{N}\sum_{i=1}^N\frac{\sum_qI\left[q\in{P}_i(t)\bigwedge q\in{T}_i\right]}{\sum_qI\left[q\in{T}_i\right]}. \end{equation*}

Here, *N* is the total number of proteins in the dataset; *M*(*t*) is the number of proteins with at least one predicted GO term with prediction score ≥ *t*. ${T}_i$ is the set of experimentally determined (ground truth) GO terms, including their parent terms, for protein *i*. ${P}_i(t)$ is the set of predicted GO terms for protein *i* with prediction score ≥ *t*. *I*[] is the standard indicator function (i.e. the Iverson’s bracket). For both ${T}_i$ and ${P}_i(t)$, the root terms of the three GO aspects (GO:0003674 ‘molecular_function’, GO:0008150 ‘biological_process’, and GO:0005575 ‘cellular_component’) are excluded.

In lay terms, precision [*pr*(*t*)] quantifies the portion of correctly predicted GO terms among all terms predicted for the target proteins. Recall [*re*(*t*)] quantifies the portion of correctly predicted GO terms among all ground truth terms that are annotated to the target proteins in the database.

The other evaluation metric used here is wFmax, which is the main evaluation metric for the currently-in-progress CAFA5 experiment. It is defined as


(15) \begin{equation*} wFmax=\underset{t\in \left(0,1\right]}{\max}\left(\frac{2\cdot wpr(t)\cdot wre(t)}{wpr(t)+ wre(t)}\right). \end{equation*}

Here, w*pr*(*t*) and w*re*(*t*) are the precision and recall, respectively, weighted by the information contents [34] of GO terms. They are defined as


(16) \begin{equation*} wpr(t)=\frac{1}{M(t)}\sum_{i=1}^{M(t)}\frac{\sum_qI\left[q\in{P}_i(t)\bigwedge q\in{T}_i\right]\cdotp IC(q)}{\sum_qI\left[q\in{P}_i(t)\right]\cdotp IC(q)} \end{equation*}

(17) \begin{equation*} wre(t)=\frac{1}{N}\sum_{i=1}^N\frac{\sum_qI\left[q\in{P}_i(t)\bigwedge q\in{T}_i\right]\cdotp IC(q)}{\sum_qI\left[q\in{T}_i\right]\cdotp IC(q)}. \end{equation*}

The information content, also known as the information accretion, for GO term *q* is defined as


(18) \begin{equation*} IC(q)=-{\mathit{\log}}_2\left(\frac{1+C(q)}{1+C\left( parent(q)\right)}\right). \end{equation*}

Here, *C*(*q*) is the number of proteins with GO terms q in the whole template database; *C*(*parent(q)*) is the number of database proteins with all parent terms of GO term *q*.

The three aspects of GO—Molecular Function (MF), Biological Process (BP), and Cellular Component (CC)—are evaluated separately. Here, MF describes the molecular activity, such as catalysis or transport, performed by a protein. BP indicates the biological pathways and developmental events that the protein is involved in. CC is the subcellular location or the protein-containing complex where the protein can be found.

## Results

### Datasets

We benchmarked different homology-based function prediction schemes on a time-elapsed test set of 4303 proteins. Each of these proteins has been annotated with at least one new GO term in any one of the three GO aspects in UniProt Gene Ontology Annotation (UniProt-GOA) release 12 July 2023 but has no GO annotation in the same aspect in UniProt-GOA release 17 November 2022. The template sequence database used by the evaluated search tools consists of 134 862 proteins with GO annotations in UniProt-GOA 17 November 2022. In line with the protocols used in recent CAFA challenges, only GO annotations with experimental or high-throughput evidence (evidence codes: EXP, IDA, IPI, IMP, IGI, IEP, HTP, HDA, HMP, HGI, HEP), traceable author statements (evidence code: TAS), or inferences made by curators (evidence code: IC), along with all their parent GO terms, are considered in curating the template database and test set. Proteins whose only leaf MF GO term is GO:0005515 ‘protein binding’ are not included as MF test protein or MF template protein. In total, the test set comprises 1119 proteins for the MF aspect, 1609 for the BP aspect, and 2468 for the CC aspect (Table S1).

### Scoring functions are critical for function prediction

**Figure 1:**
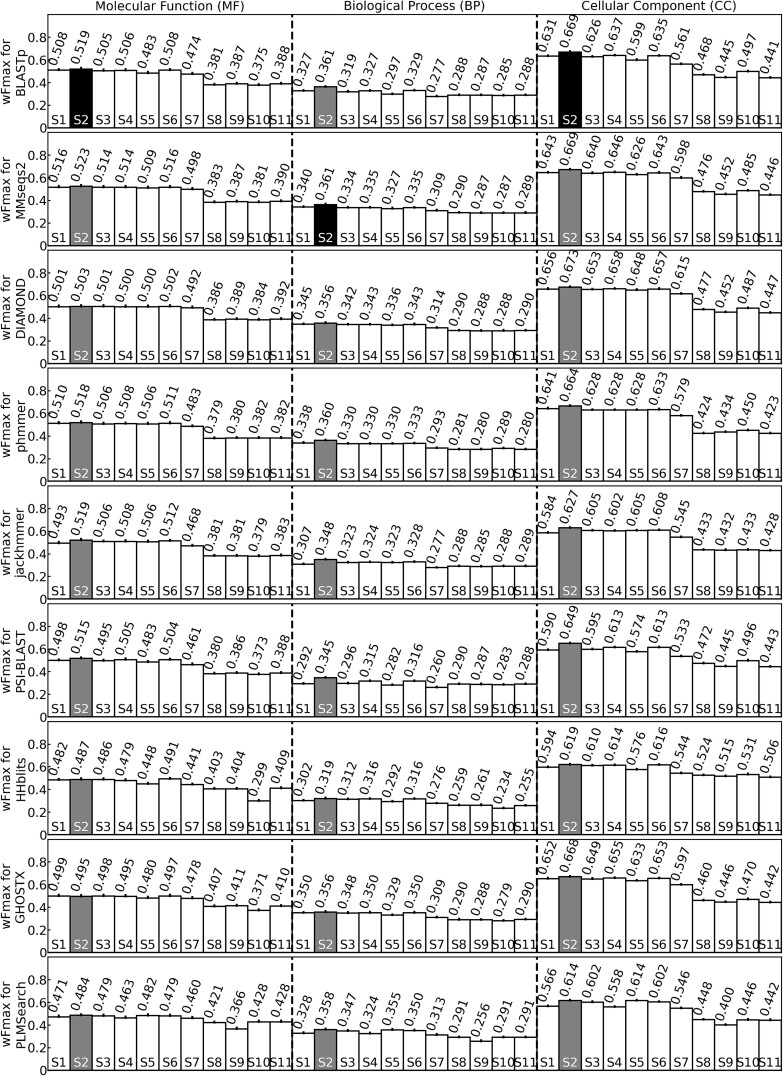
The wFmax values for GO prediction; the wFmax values range between 0 and 1, where a higher value indicates a more accurate prediction, and each row represents a different database search tool, which different bars within each row correspond to the 11 scoring functions, and each of the three columns corresponds to one of the three aspects of the GO hierarchy. The lengths of the error bars are equal to the standard error of mean (SEM) of weighted *F*-measure values per protein; gray bars indicate the highest wFmax value for each method; black bars indicate the highest wFmax value among all methods for a particular GO aspect; the ns, ^*^, ^*^^*^, and ^*^^*^^*^ signs mean .05 < *P*-value, .01 < *P*-value ≤ .05, .001 < *P*-value ≤ .01, and *P*-value ≤ .001, respectively, where *P*-values are calculated by two-tailed paired *t*-test for *F*-measure values per protein between the most accurate scoring function (*S*_2_) and another scoring function; since PLMSearch reports cosine (cos)-similarity instead of bit-score, the cos-similarity is used to calculate *S*_1_ and *S*_2_ for PLMSearch; following the suggestion from the original PLMSearch paper, only the top 10 hits with the highest cos-similarity from the PLMSearch results are used.

Our large-scale benchmarks reveal that even when utilizing the same search database tool, varying scoring functions can drastically influence the performance of homology-based protein function prediction (Figs 1 and S1). For instance, when using BLASTp, the top-performing scoring function (*S*_2_) exhibits wFmax values higher by 33.3%, 83.4%, and 15.2% than the worst scoring functions for MF, BP, and CC, respectively.

Independent of sequence database search tools and GO aspects, the scoring function *S*_2_, which weighs all templates by both bit-scores and sequence identities, performs the best (black bars in Figs 1 and S1, see online supplementary material for a color version of this figure). This is followed by functions *S*_1_ and *S*_3_ to *S*_6_, which weigh all templates either by bit-scores or sequence identities, but not both. For MF and CC, the worst scoring functions are those that solely consider the template with the highest sequence identity (*S*_8_ to *S*_11_). On the other hand, the scoring function that considers the frequency of a GO term among all templates (*S*_7_) outperforms *S*_8_ to *S*_11_ but still underperforms when compared with *S*_1_ to *S*_6_. For BP, functions *S*_7_ to *S*_11_ exhibit poor performance at similar levels. The better performance of *S*_2_ compared with all other scoring function is probably because it is the only score that considers both length-dependent (bit-score) and length-independent (sequence identity) metrics for sequence similarities between the query and all templates. Furthermore, we found that although *S*_2_ is partly based on the length-dependent bit-score, its good performance compared with other scoring functions is consistent across target proteins of almost any length and for any of the three GO aspects (Fig. S2, see online supplementary material for a color version of this figure). We also found that although proteins with more specific GO terms (i.e. GO terms with higher information content) are more difficult to predict, *S*_2_ maintains its advantage over other scoring functions across almost all ranges of GO term information content (Fig. S3, see online supplementary material for a color version of this figure).

While *S*_2_ clearly stands out as the most suitable individual scoring function for function prediction among all 11 scoring functions considered, we wondered if a combination of *S*_2_ with another scoring function could further improves function prediction. We evaluated the linear combination $\lambda{S}_2-\left(1-\lambda \right){S}_c$, where $\lambda \in \left[0,1\right]$ is the weight between *S*_2_ and another scoring function (Fig. S4, see online supplementary material for a color version of this figure), for each other scoring function *c*. For MF and CC, no combination can improve BLASTp-based GO prediction accuracy relative to using *S*_2_ alone (i.e. $\lambda =1$). On the other hand, while combining *S*_2_ with *S*_1_, *S*_3_, *S*_4_, *S*_5_, *S*_6_, or *S*_7_ cannot improve BP prediction, combing *S*_2_ with *S*_8_, *S*_9_, *S*_10_, or *S*_11_ can improve BP prediction accuracy but only slightly. For example, the highest BP prediction accuracy is achieved by a half-half combination of *S*_2_ and *S*_11_ (wFmax = 0.368), which is only marginally higher than *S*_2_ alone (wFmax = 0.361). Therefore, for simplicity, the following sections only consider *S*_2_.

Perhaps surprisingly, a comparison with recent deep learning models [11–13, 15, 35, 36] for GO prediction shows that BLASTp with *S*_2_ is better many deep learning models, including DeepGO-SE [35], DeepGOplus [11], ProteInfer [15], AnnoPro [36], and TALE [12] (Fig. S5). This shows that sequence homology is still an important component of protein function prediction even in the era of deep learning.

### Traditional sequence-sequence alignment outperforms hidden Markov model for function prediction

Our previous study [24] demonstrated that more sensitive HMM-based sequence searches, particularly those by HHblits, markedly enhanced protein structure prediction compared with sequence-sequence alignment. However, the opposite holds true for protein function prediction (Fig. 1). In fact, HMM-based sequence search tools such as jackhmmer and HHblits generally perform worse than sequence-sequence alignment-based tools such as BLASTp and MMseqs2 almost across all scoring functions—with HHblits trailing as the least effective sequence search tool in nearly all scenarios. This discrepancy might be attributed to the optimization of HHblits for structure prediction tasks, including remote structure analog detection [28]. An exception to these discoveries is GHOSTX, which performs traditional sequence-sequence alignment but was worse than some HMM-based tools such as phmmer for protein function prediction. This is likely because GHOSTX was mainly optimized for translated nucleotide database searches [23] and not for protein–protein sequence alignment.

We notice that programs employing iterative database search, e.g. PSI-BLAST and jackhmmer, tend to have worse GO prediction accuracies compared with similar programs using less sensitive non-iterative modes (BLASTp and phmmer, respectively, Fig. 1). Similarly, for MMseqs2, we observe a minor but consistent decrease in GO prediction accuracies with an increase in the number of iterations (Fig. S6, see online supplementary material for a color version of this figure). This finding contrasts with the case in protein structure prediction tasks [24], where iterative searches typically enhance structure modeling quality, presumably by providing deeper and more informative MSAs. The worse performance of iterative search tools for homology-based function prediction is probably caused by profile drift, i.e. more distantly related sequences with similar structure but dissimilar functions are progressively included with increasing number of iterations, as shown below in a case study on the TMTC4 protein.

We also found that the protein language model-based sequence search tool, PLMSearch, has the worst performance in MF and CC among all sequence search tools in this benchmark (Figs 1 and S1). This is different from the results in the original PLMSearch study [29], which shows that PLMSearch has higher sensitivity in detecting template proteins with similar structures than BLASTp, MMseqs2, and HHblits; the discrepancy likely arises simply due to the different requirements of the tasks of structural versus functional templates.

All these findings unanimously suggest that function prediction necessitates less sensitivity in template detection than structure prediction. This result is not surprising, given previous studies which indicated that even though proteins sharing a sequence identity as low as 30% [37] often exhibit similar structures, a sequence identity of at least 50% [38, 39] to 60% [40] must be maintained to ensure similar biological functions. Indeed, more remote homologs may contaminate function predictions with low-confidence information, thus decreasing performance.

We also wondered whether HMM-based methods have greater advantages for annotation of harder targets with fewer homologous templates. To this end, we partitioned the benchmark dataset into three subgroups: limited knowledge targets that have a GO annotation in a different GO aspect in the template database; no knowledge (NK) easy targets that do not have prior GO annotation but have a function template with >50% sequence identity; and no knowledge (NK) hard targets without any function templates with >50% sequence identity. Although some HMM-based methods slightly outperform BLASTp for MF (HHblits and jackhmmer), BP (HHblits), and CC (phmmer) for the most challenging NK-hard targets, the difference between BLASTp and the most accurate HMM-based methods in each category is small in magnitude and is not found to be statistically significant (paired *t*-test *P*-values > .05, Fig. S7). Moreover, combining BLASTp and phmmer, which is overall the most accurate HMM method for GO prediction (Fig. 1), leads to no improvement to BP and CC prediction and a 1.3% improvement for MF (*P*-value = 1.41E−2, Fig. S8, see online supplementary material for a color version of this figure). Since this improvement is only marginally significant, the following section does not consider the combination of two sequence search tools.

Overall, the best GO prediction results are produced by MMseqs2 for MF, by both BLASTp and MMseqs2 for BP, and by DIAMOND for CC. Consequently, the following section will concentrate on these three programs while using the uniformly optimal scoring function (*S*_2_).

### Proper parameter settings improve DIAMOND-based function prediction

Among all sequence search tools reviewed by this work, only BLASTp and PSI-BLAST were originally developed with detection of functionally related homologs in mind. Both BLAST and PSI-BLAST were optimized to recognized homologs from the same protein family with related functions such as the immunoglobins [41] and BRCT proteins involved in DNA damage-responsive cell cycle checkpoints [19]. The remaining tools are optimized either for the recognition of analogous proteins with similar structures but not necessarily similar functions (DIAMOND, MMseqs2, jackhammer, phmmer, and HHblits) or for translated nucleotide sequence search (GHOSTX). Therefore, the search parameters of these search tools may not be optimized for function prediction.

For example, while BLASTp, DIAMOND, and MMseqs2 all perform non-iterative sequence-sequence searches, their default settings differ greatly. For instance, the default *E*-value cutoffs for BLASTp and DIAMOND are 10 and 0.001, respectively, while the default number of top hits are 500 and 25. Moreover, both DIAMOND and MMseqs2 can function under different sensitivity modes. Therefore, we examined how varying search parameters, such as *E*-value cutoffs, sensitivity modes, and the maximum number of top hits, impact the effectiveness of function prediction.

**Figure 2:**
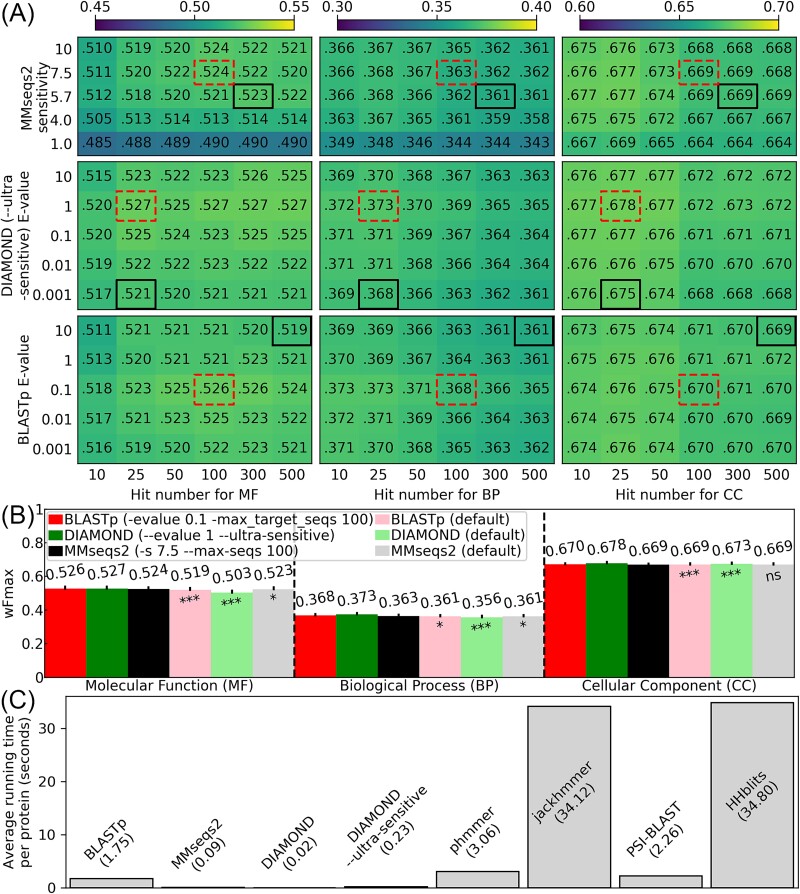
Parameter optimization for GO prediction using scoring function *S*_2_; (A) Heatmap for wFmax values using different *E*-values/sensitivities and maximum hit number, and black solid boxes and red dashed boxes indicate the default and optimized parameters, respectively; (B) the wFmax values for the optimal parameters (dark color bars) and default parameters (light color bars) for each tool, and the error bar lengths reflect the SEM of weighted *F*-measure values per protein; the ns, ^*^, and ^*^^*^^*^ signs means .05 < *P*-value, .01 < *P*-value ≤ .05, and *P*-value ≤ .001, respectively, where *P*-values are calculated by two-tailed paired *t*-test for *F*-measure values per protein between the optimal parameters and default parameters; (C) average running time of different sequence search tools; since DIAMOND running at the default sensitivity mode and ultra-sensitive mode have very different speeds, the running times of both are shown.

After assessing various parameter combinations, we discovered that using non-default settings on MMseqs2 only improves function prediction accuracy very slightly (Figs 2 and S9, see online supplementary material for a color version of this figure). Conversely, operating BLASTp at a lower *E*-value cutoff (-evalue 0.1) and reducing the number of hits (-max_target_seqs 100) mildly improves MF, BP, and CC prediction wFmax by 1.3%, 1.9%, and 0.1%, respectively (Figs 2 and S10, see online supplementary material for a color version of this figure). DIAMOND benefits the most from parameter tuning, which improves wFmax of MF, BP, and CC prediction by 4.8%, 4.8%, and 0.7%, respectively (Fig. 2B). Higher sensitivity settings (--ultra-sensitive, --very-sensitive, or --more-sensitive) generally enhance GO prediction accuracies (Figs S11 and S12). Moreover, a lenient *E*-value cutoff (--evalue 1) also boosts prediction accuracies (Fig. S13, see online supplementary material for a color version of this figure). Here, an improvement of 0.7% for DIAMOND-based CC prediction is not only statistically significant (paired *t*-test *P*-value 1.62e−6, Fig. 2B) but also biologically relevant. For example, on average, an experimentally annotated protein has 13 CC terms (including parent terms); the human genome has around 20 000 protein coding genes. Thus, a 0.7% difference in wFmax would mean ~2000 GO terms being predicted differently for the human genome.

Despite DIAMOND performing less effectively than MMseqs2 and BLASTp for GO prediction when all programs operate under their default settings, it performs comparably or slightly better than the other two programs when all programs operate with the optimal search parameters (‘--evalue 1 --ultra-sensitive’ for DIAMOND, ‘-evalue 0.1 -max_target_seqs 100’ for BLASTp, and ‘-s 7.5 --max-seqs 100’ for MMseqs2, Fig. 2). Specifically, after adjusting search parameters, DIAMOND and BLASTp have comparable MF and BP accuracies, which are superior to that of MMseqs2. With parameter tuning, DIAMOND’s wFmax values for CC show enhancements of 1.2% over those from BLASTp, which, in turn, outperforms MMseqs2. These adjustments position DIAMOND as an attractive choice for large-scale function prediction, particularly considering its >7 times faster speed compared with BLASTp (Fig. 2C).

To provide an additional test of our methods on a data set completely separated from our testing to this point, we repeated the same experiment using the CAFA3 dataset (Fig. S14, see online supplementary material for a color version of this figure) and the same set of optimized parameters obtained in Fig. 2B. The test result on this smaller dataset is largely consistent with the findings above: the GO prediction performance by BLASTp and MMseqs2 is comparable between the default parameters and the optimized parameters. On the other hand, the performance of DIAMOND can be significantly improved by parameter tuning: DIAMOND run under the default setting lags far behind BLASTp and MMseqs2 on all GO aspects, but DIAMOND is comparable to the other program when running at a higher sensitivity mode.

### Case study: function prediction of *Drosophila melanogaster* TMTC4

**Figure 3:**
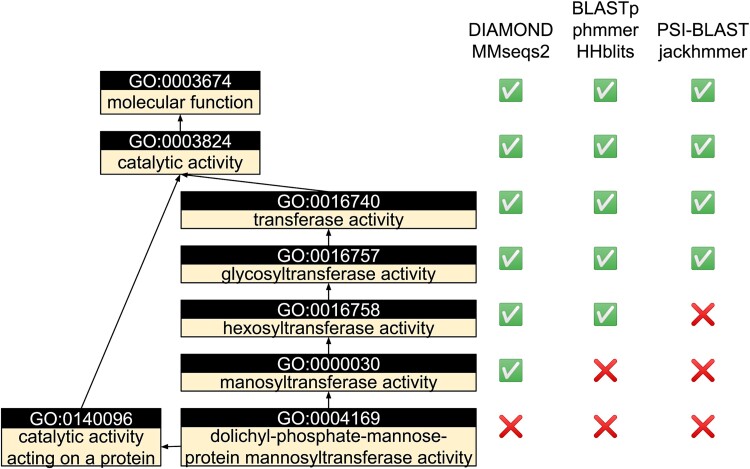
MF GO annotation for TMTC4 (UniProt accession: Q9VF81). Ground truth annotations are shown on the left; whether or not GO prediction from different search tool contains the correct GO term are marked by checks (correct) and crosses (missing) in the table on the right.

To delve further into the impact of different sequence search tools on function prediction, we use the TMTC4 protein from the fruit fly (UniProt accession: Q9VF81) as a case study. This protein is a dolichyl-phosphate-mannose-protein mannosyltransferase (GO:0004169) [42] (Fig. 3). While none of the programs can predict this highly specific MF GO term (Fig. 3), all of them can predict some parent terms of this GO term. DIAMOND and MMseqs2 provide the most specific parent term, GO:0000030 ‘mannosyltransferase activity’ (weighted *F*-measure [wF] = 0.860). Next in line is BLASTp (wF = 0.706), followed by HHblits and phmmer (wF = 0.664) predicting its parent term, GO:0016758 ‘hexosyltransferase activity’. The least specific predictions come from PSI-BLAST and jackhmmer (wF = 0.628), which merely predict an even less specific parent term, GO:0016757 ‘glycosyltransferase activity’.

The inferior performance of the iterative searching tools (PSI-BLAST and jackhmmer) relative to their respective non-iterative counterparts (BLASTp and phmmer) primarily results from incorporating functionally less relevant templates during the iterations. For instance, in jackhmmer’s first iteration, 107 out of 344 (31.1%) hits are annotated with the correct parent term, GO:0016758. This ratio decreases to 89 out of 1273 (7.0%) and 75 out of 1767 (4.2%) hits in the second and final iterations, respectively. Consequently, the prediction score of GO:0016758 plunges from 0.777 in the first iteration to 0.460 in the final iteration. This phenomenon, known as ‘profile drift’ [43], implies that an increased number of iterations progressively change the composition of the query sequence profile, leading to the inclusion of very distant sequence relatives in the search results.

### Case study: function prediction of *Danio rerio* ER[b]1

**Figure 4:**
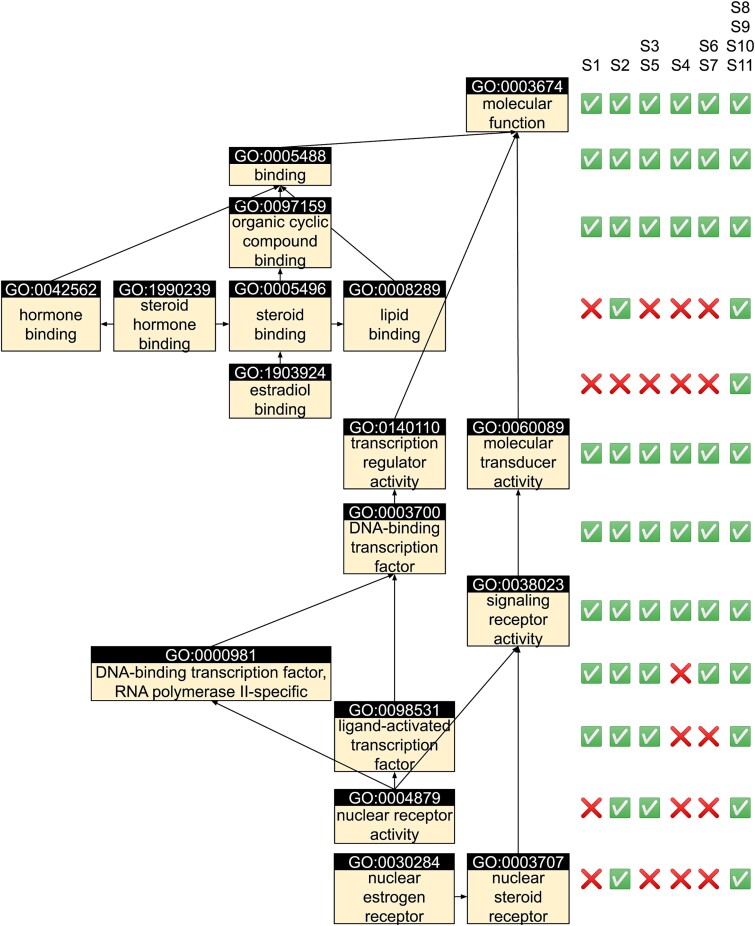
MF GO annotation for ER[b]1 (UniProt accession: A0A8M2B359); ground truth annotations are shown on the left; whether or not GO prediction from different search tool contains the correct GO term are marked by checks (correct) and crosses (missing) in the table on the right.

To better understand the impact of using different scoring functions on function prediction, we use the BLASTp prediction for ER[b]1 protein from zebra fish (UniProt accession: A0A8M2B359) as a case study. ER[b]1 is an estrogen receptor located at the nucleus [44–46]. It has the following leaf MF terms: GO:1903924 ‘estradiol binding’, GO:1990239 ‘steroid hormone binding’, GO:0004879 ‘nuclear receptor activity’, and GO:0030284 ‘nuclear estrogen receptor’ (Fig. 4). This target protein has many sequence homologs (301 templates with MF annotation in a default BLASTp run) and is therefore a relatively easy to predict target.

Although all four scoring functions that use the maximum sequence identity (*S*_8_, *S*_9_, *S*_10_, and *S*_11_) can predict all four leaf MF terms, their weighted *F*-measure is all low (wF = 0.363, 0.358, 0.367, and 0.363, respectively). This is because they also have many false positive predictions such as GO:0001093 ‘TFIIB-class transcription factor binding’, GO:0036312 ‘phosphatidylinositol 3-kinase regulatory subunit binding’, and GO:0031798 ‘type 1 metabotropic glutamate receptor binding’. All these unrelated terms are contributed by only 1 out of 301 templates and are not predicted by scoring functions that consider a consensus of all templates (*S*_1_ to *S*_7_).

On the other hand, scoring functions *S*_1_ to *S*_7_ has far fewer false positive hits and therefore much better wF > 0.4. Among them, the most accurate prediction is achieved by *S*_2_ (wF = 0.958), which predicts GO:1990239 ‘steroid hormone binding’, GO:0004879 ‘nuclear receptor activity’, and GO:0030284 ‘nuclear estrogen receptor’, while the second best prediction (*S*_1_, wF = 0.563) can only predict their parent terms GO:0097159 ‘organic cyclic compound binding’, GO:00098531 ‘ligand-activated transcription factor activity’, and GO:0038023 ‘signaling receptor activities’. This discrepancy is mainly caused by the different template weighting schemes implemented by different scoring functions. The advantages of S_2_ are particularly important for this target because only 14 out of all 301 template hits have all the leaf terms of the target. Therefore, scoring functions that do not differentiate relevant versus irrelevant templates well enough (e.g. *S*_7_ which weight all templates equally, wF = 0.433) perform poorly. While the 14 most relevant templates have on average both higher bit-scores (673.4 versus 142.3) and higher sequence identities (72.1% versus 49.5%) than the remaining 287 templates, the latter set of templates still have a large impact on the consensus prediction due to their larger number. Only *S*_2_, which weights templates by both bit-scores and sequence identities, weights the most relevant templates heavy enough to make the most accurate prediction.

## Conclusions

In this study, we evaluated the usefulness of several commonly used sequence search tools for protein function prediction. We discovered that, despite DIAMOND being less effective than BLASTp and MMseqs2 for GO prediction under default parameter settings, it can surpass both programs in terms of GO prediction accuracies merely by adjusting the sensitivity and *E*-value cutoff settings. These three methods demonstrate higher GO prediction accuracies compared with more sensitive sequence search protocols, including PSI-BLAST, HHblits, jackhmmer, and phmmer, all of which are based on HMMs, iterative search, or both. Alongside evaluating different search tools, this study reaffirms previous findings that GO predictions stemming from multiple templates are more accurate than those derived from the template with the highest sequence identity. We also identified a new scoring function (*S*_2_) that consistently outshines the extensively employed DiamondScore (*S*_1_) scoring function featured in many function prediction programs [11–13, 17, 18].

While there are many factors that affect the accuracy of protein function prediction such as feature selection and integration of multiple sources of information, this study focuses solely on exploring the best practice on sequence homology-based function prediction. In another study, we integrated BLASTp with *S*_2_ into our latest protein function predictor, StarFunc [47], which combines multiple sources of information including sequence homology, structure templates, protein–protein interactions, protein families, and deep learning. The newly discovered *S*_2_ scoring function has made an important contribution to the overall performance of StarFunc, which was the 5th ranked model among 1625 teams from 96 countries in last year’s CAFA5 challenge for automated protein function annotation. We expect that our scoring function optimizations would also prove beneficial for other similar workflows.

In this study, we did not fully explore whether the combination of multiple sequence search tools could yield further improved function prediction accuracies. This remains a valuable topic for future investigation, as previous studies from our group [8, 9] and others [6] as well as new benchmark data in this study (Fig. S6, see online supplementary material for a color version of this figure) indicate that combining two different sequence search tools can achieve slightly better result than using either search tool alone.

**Key Points**

- Under the default settings, BLASTp and MMseqs consistently outperform DIAMOND, jackhmmer, phmmer, HHblits, PSI-BLAST, GHOSTX, and PLMSearch for protein function prediction.
- Proper parameter setting can improve DIAMOND-based function prediction, resulting in GO prediction accuracies that rival those from BLASTp and MMseqs.
- This study found a new scoring function that consistently improves GO prediction from given set of template hits, regardless of sequence search tools.

## Data availability

Source code and data for performing all analysis of this study are available at https://github.com/kad-ecoli/blastKNN_benchmark/.

## Supplementary Material

blastKNN_SI_20240529_bbae349
