# Supplementary material for: A large-scale assessment of sequence database search tools for homology-based protein function prediction
Source: Brief Bioinform. 2024 Jul 22;25(4):bbae349. doi: 10.1093/bib/bbae349 (PMC11262835; doi:10.1093/bib/bbae349)
Supplement: blastKNN_SI_20240529_bbae349 [file blastknn_si_20240529_bbae349.docx]

**A large-scale assessment of sequence database search tools for homology-based protein function prediction**

**Supporting Information**

**Table of Content**

**Supporting Figures**

**Figure S1.** The Fmax values for GO prediction by 8 database search tools and 11 scoring functions.

**Figure S2.** The wFmax values for BLASTp-based GO prediction by different scoring functions at different sequence length bins.

**Figure S3.** The wFmax values for BLASTp-based GO prediction by different scoring functions for proteins with different maximum GO term information content.

**Figure S4.** The wFmax values for BLASTp-based GO prediction by a composite scoring function that combines *S*_2_ with another scoring function.

**Figure S5.** The wFmax values for BLASTp using *S*_2_ and recent deep learning methods for GO term prediction.

**Figure S6.** The wFmax values for GO prediction by MMseqs2 using different number of iterations and different number of hits.

**Figure S7.** The wFmax values for GO prediction by 8 database search tools using *S*_2_ on three different subsets of the benchmark dataset.

**Figure S8.** The wFmax values for GO prediction using combinations of BLASTp with other sequence search tools.

**Figure S9.** The wFmax and Fmax values for GO prediction by MMseqs2 using different sensitivities and hit numbers.

**Figure S10.** The wFmax and Fmax values for GO prediction by BLASTp using different E-value cutoffs and hit numbers.

**Figure S11.** The wFmax values for GO prediction by DIAMOND using 11 scoring functions and different sensitivity levels.

**Figure S12.** The Fmax values for GO prediction by DIAMOND using 11 scoring functions and different sensitivity levels.

**Figure S13.** The wFmax and Fmax values for GO prediction by DIAMOND using different E-value cutoffs and hit numbers.

**Figure S14.** The wFmax values for GO prediction on the CAFA3 set using the optimized parameters and default parameters for each tool.

.

**Supporting Table**

**Table S1.** Number of protein targets in different subsets of the benchmark dataset.

**
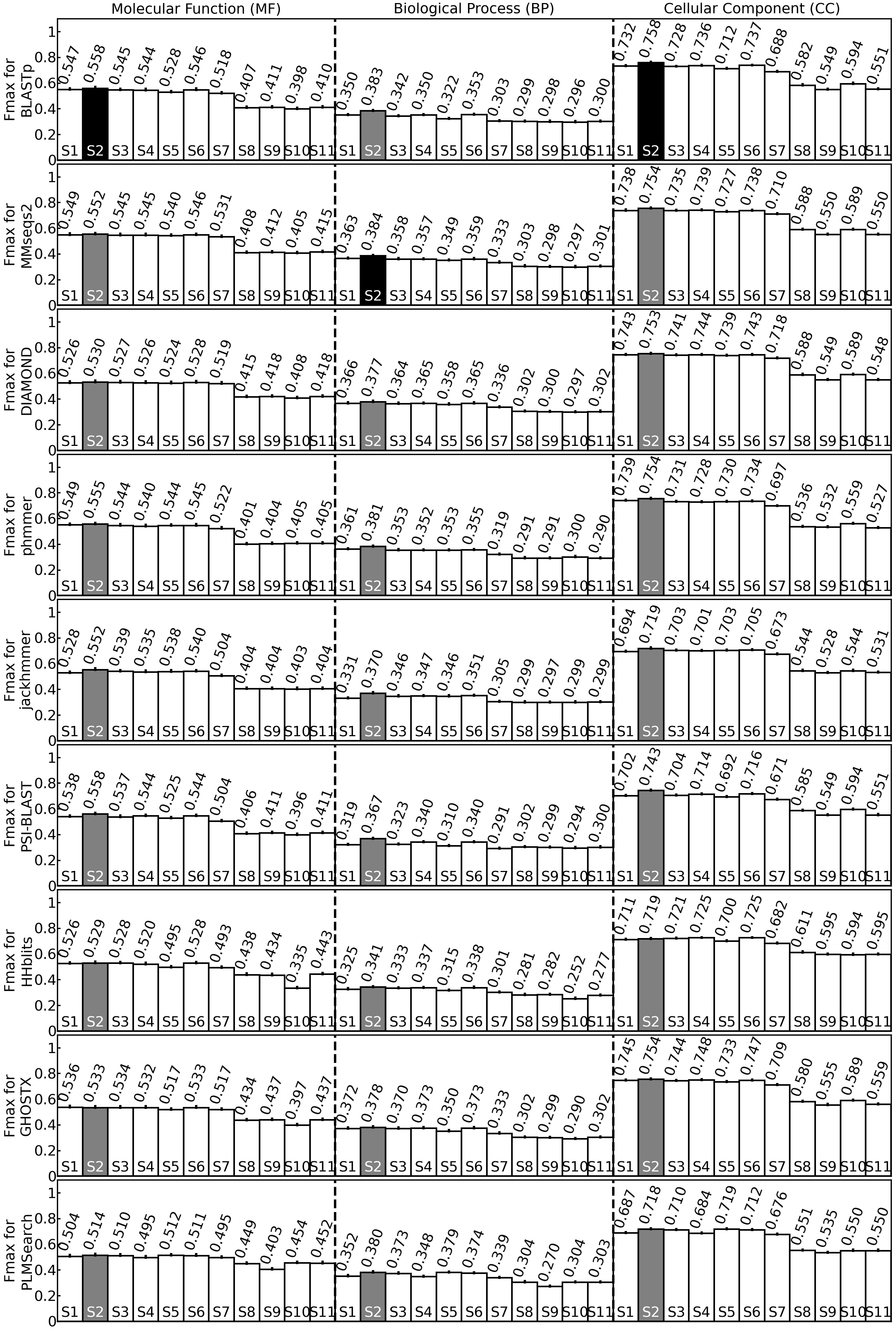
Supporting Figures**

**Figure S1.** The Fmax values for GO prediction by 8 database search tools (different rows) and 11 scoring functions (different bars in a row). Error bars indicate standard error of mean (SEM) of the per protein Fmax values. Grey bars indicate the highest Fmax value for each method. Black bars indicate the highest Fmax value among all methods.

**
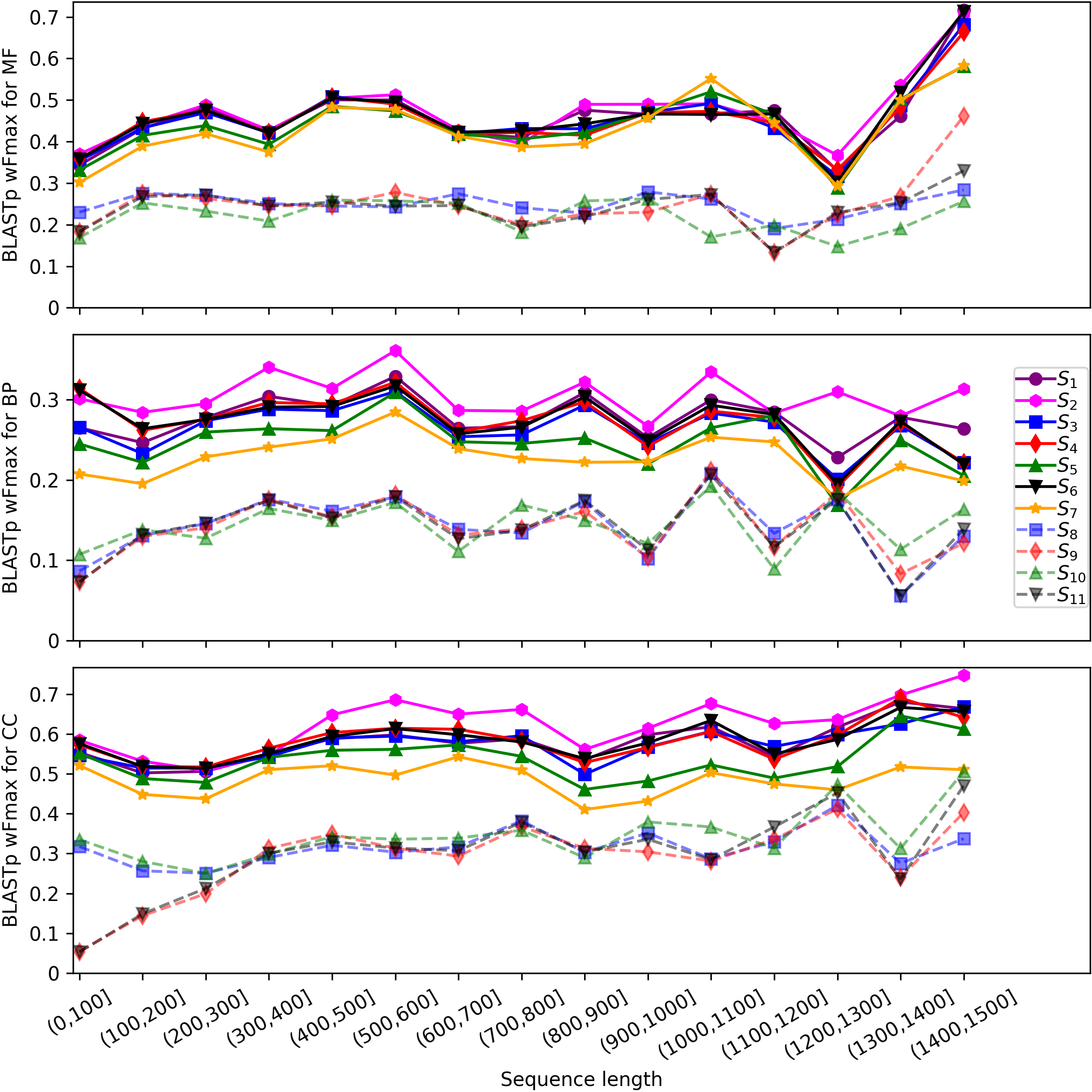
Figure S2.** The wFmax values for BLASTp-based GO prediction by different scoring functions at different sequence length bins.


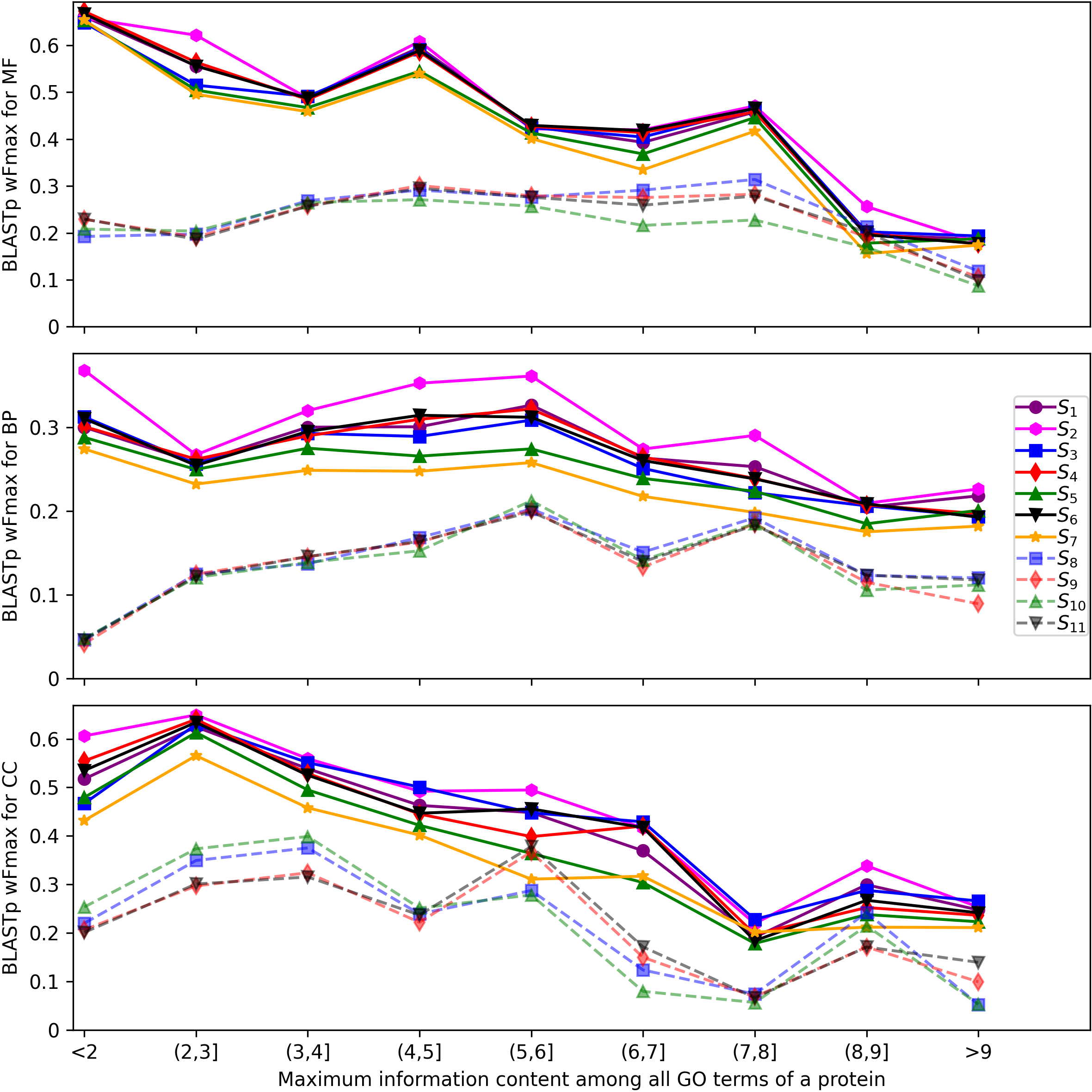
**Figure S3.** The wFmax values for BLASTp-based GO prediction by different scoring functions for proteins with different maximum GO term information content.

**
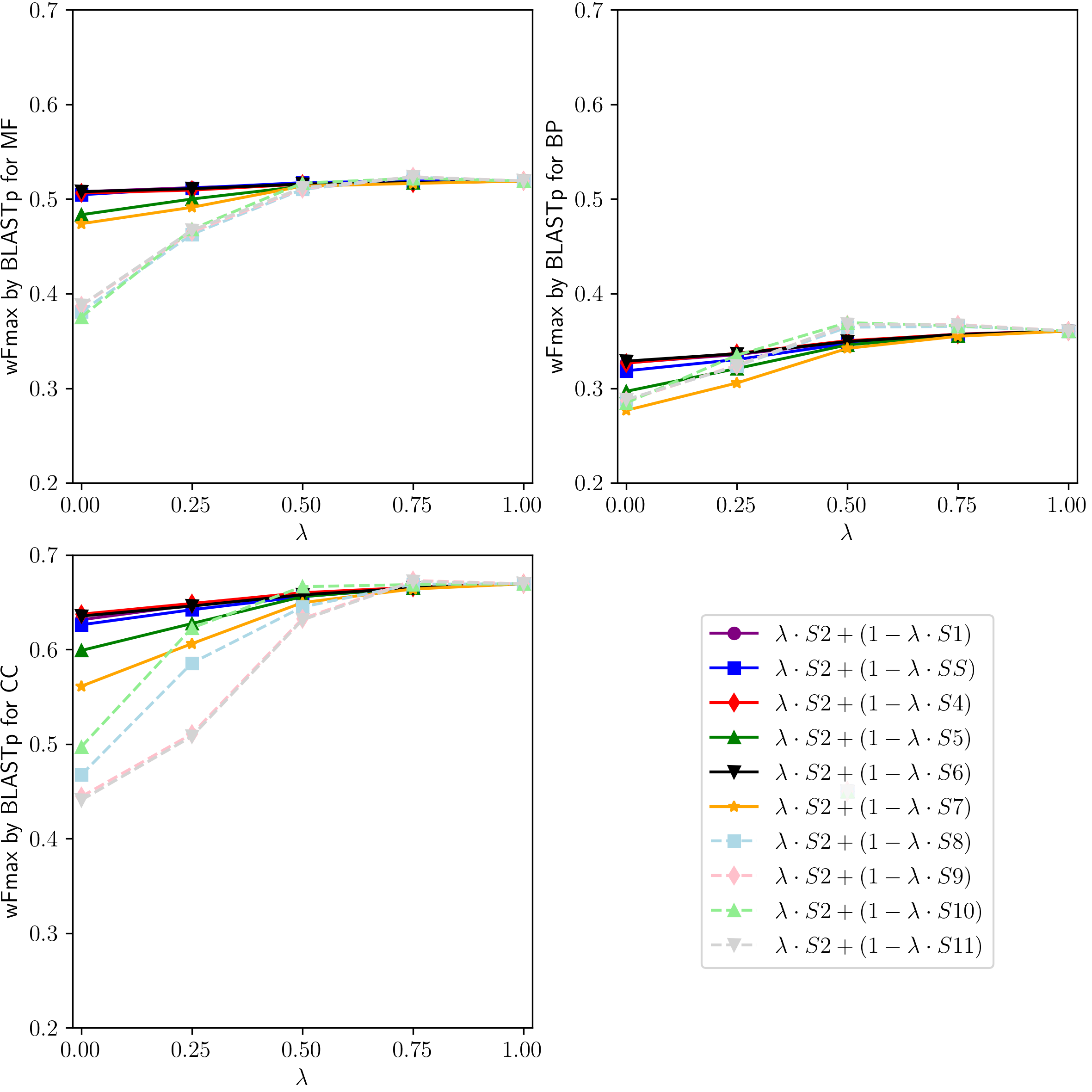
Figure S4.** The wFmax values for BLASTp-based GO prediction by a composite scoring function that combines *S*_2_ with another scoring function. When λ=1, the composite scoring function is equivalent to *S*_2_.

**
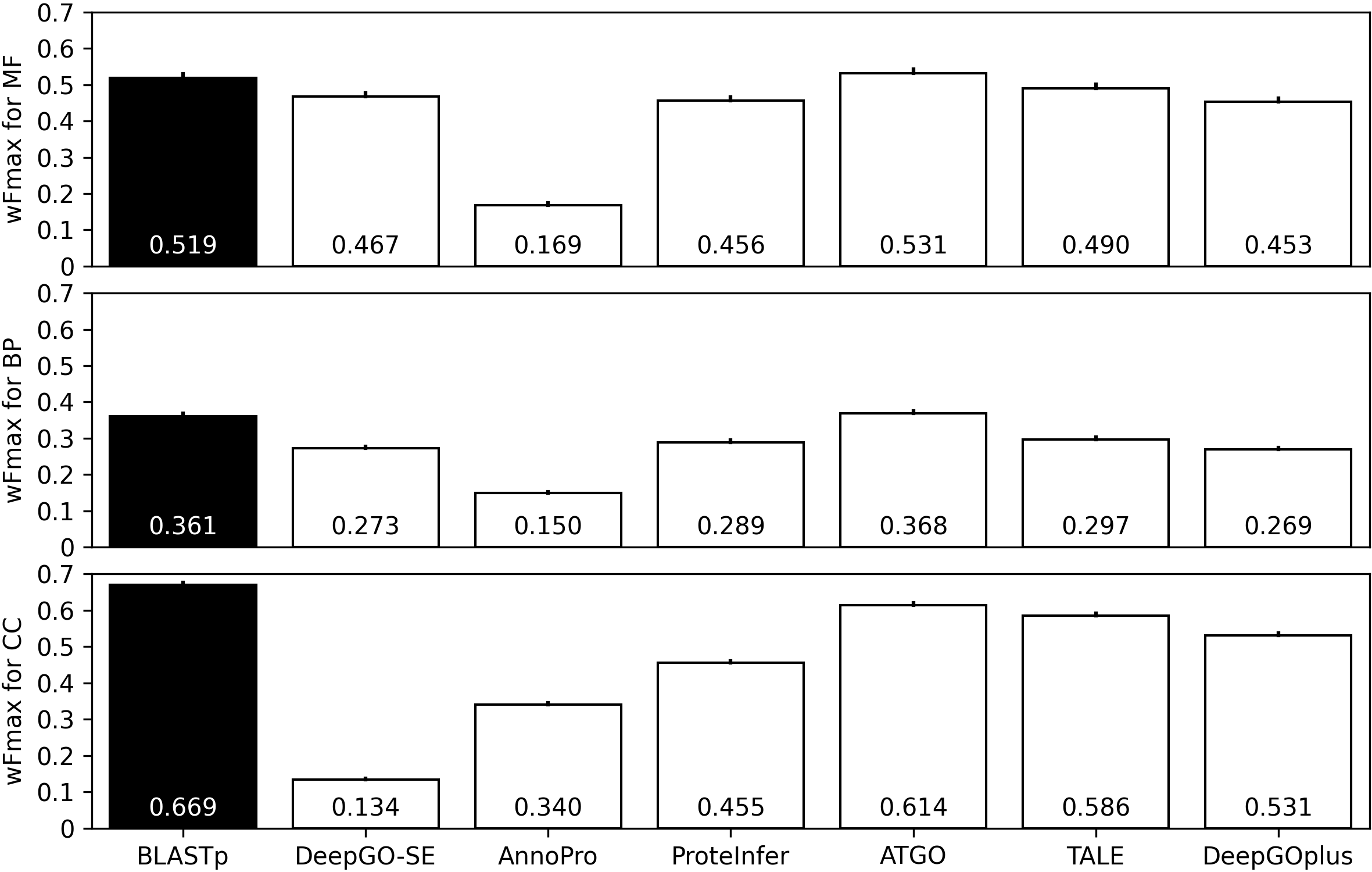
Figure S5.** The wFmax values for BLASTp using *S*_2_ and recent deep learning methods for GO term prediction. Deep learning methods are sorted in anti-chronological order. Several deep learning methods such as ATGO, TALE and AnnoPro have the option to combine deep learning-based predictions with sequence homology-based predictions. Since this benchmark aims to evaluate pure sequence homology-based method (BLASTp) can perform on par with pure deep learning methods, the option to use sequence homology search are disabled for these deep learning methods. The lengths of the error bars are equal to the standard error of mean (SEM) of weighted F-measure values per protein.

**
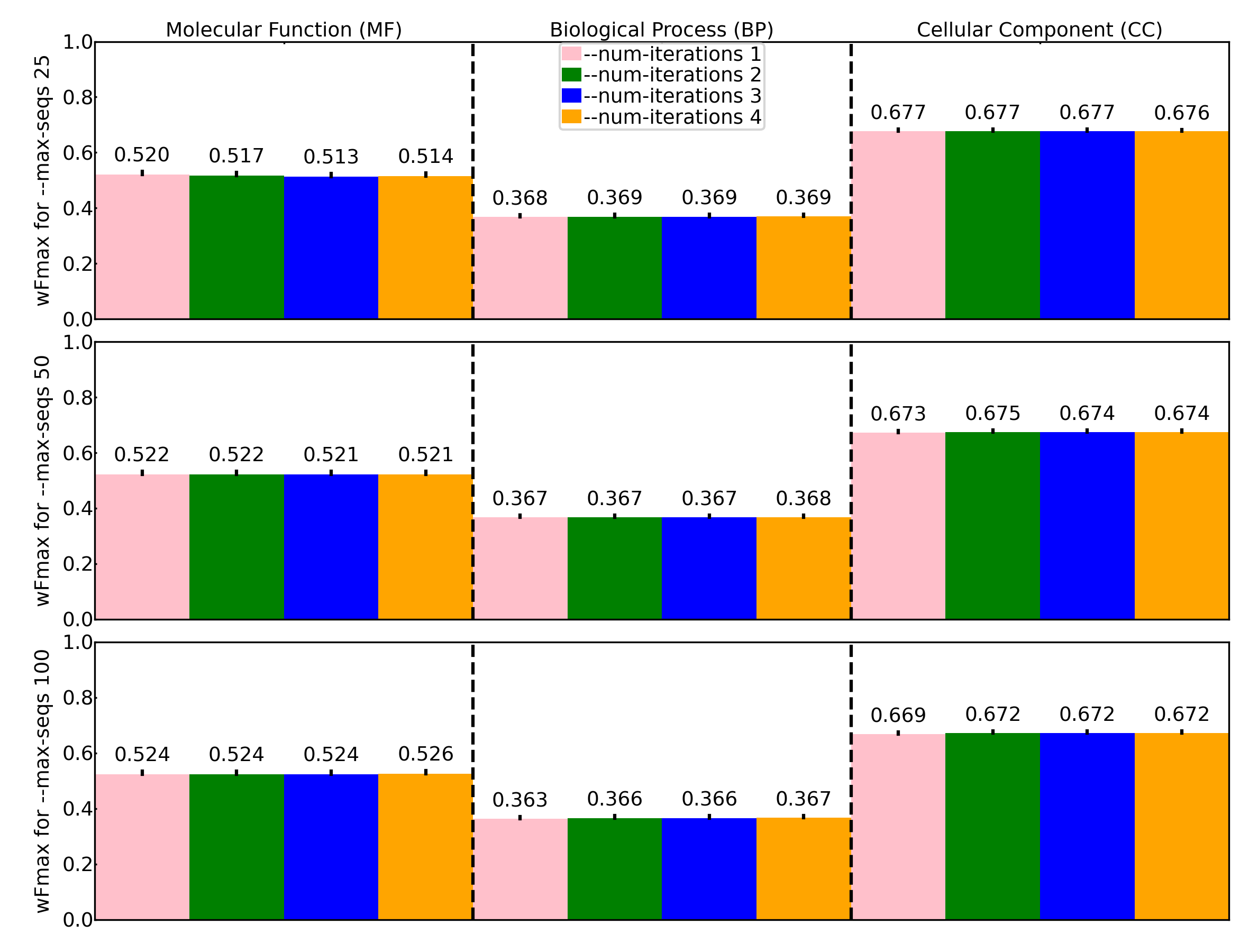
Figure S6.** The wFmax values for GO prediction by MMseqs2 using different number of iterations (different bars) and different numbers of hits (different rows). The lengths of the error bars equal to the standard error of mean (SEM) of the per protein wFmax values.

**
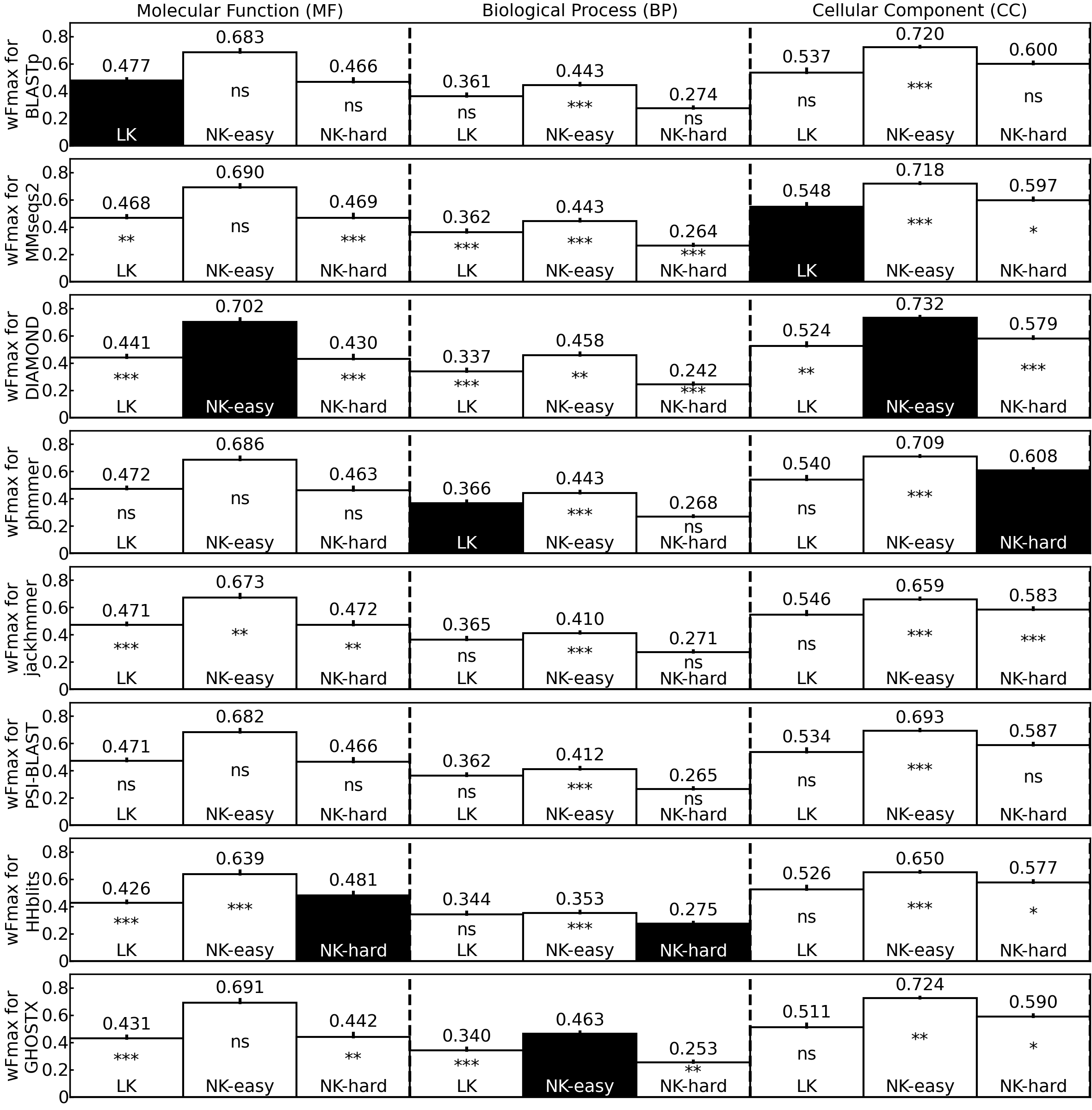
Figure S7.** The wFmax values for GO prediction by 8 database search tools using scoring function *S*_2_ on three different subsets of the benchmark dataset. Limited knowledge (LK) targets have a GO annotation in a different GO aspect in the template database. No knowledge (NK) targets do not have prior GO annotations. NK-easy and NK-hard targets are NK targets with and without function template sharing >50% sequence identity, respectively.


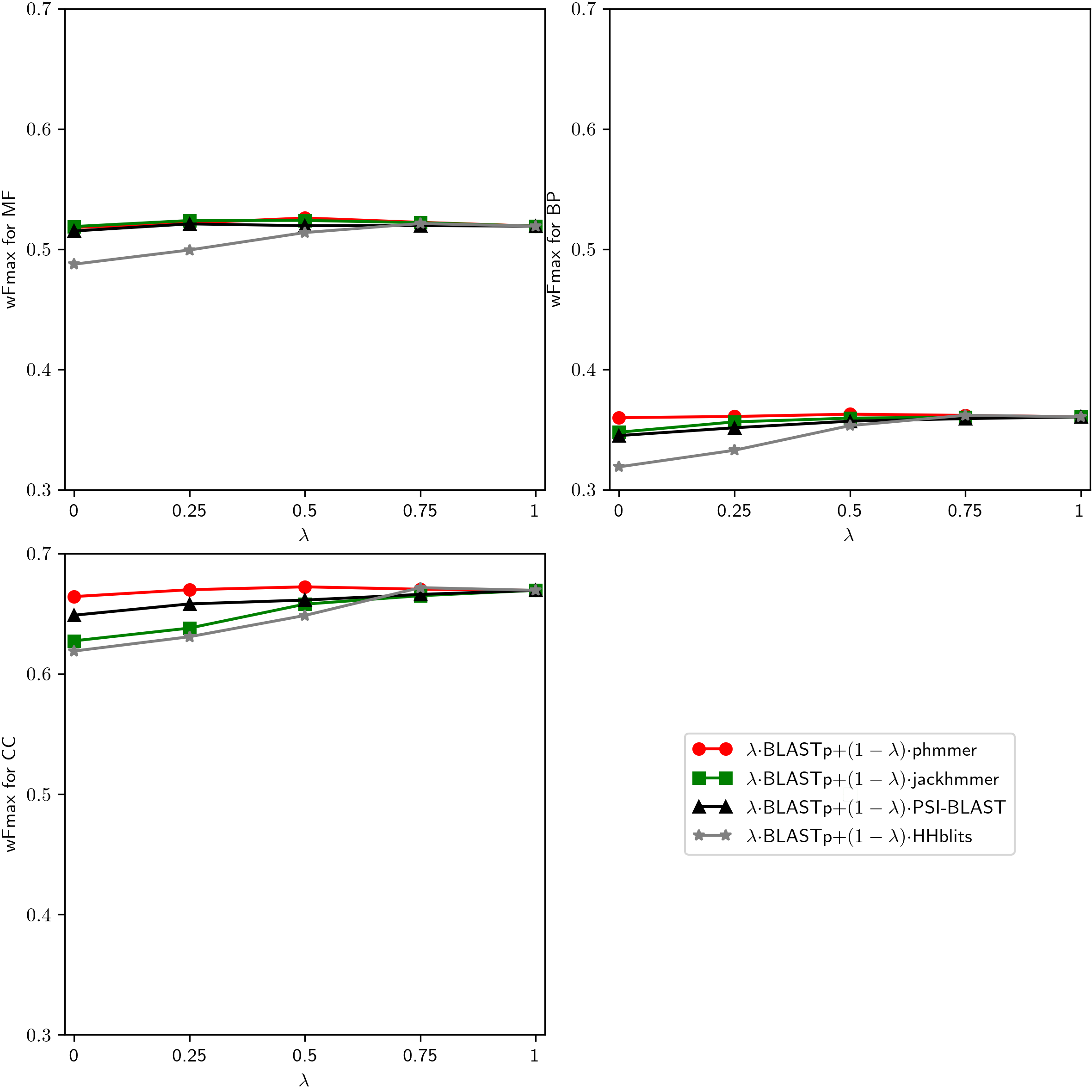


**Figure S8.** The wFmax values for GO prediction by linear combination of BLASTp and another sequence search tool, both using scoring function *S*_2_. λ is the weight between BLASTp and the other sequence search tool. λ=1 is equivalent to using BLASTp alone.


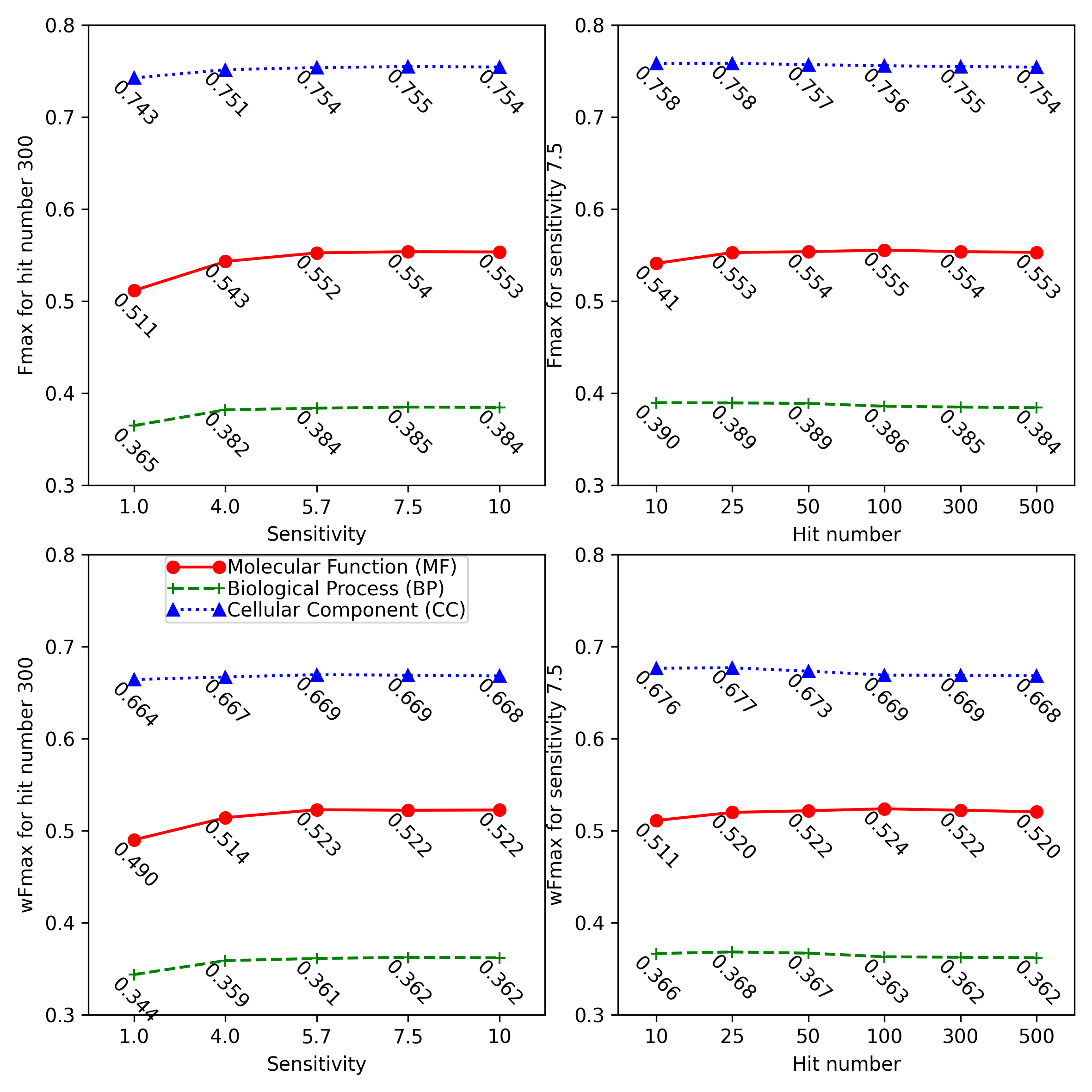
**Figure S9.** The wFmax and Fmax values for GO prediction by MMseqs2 using different sensitivities (-s) and hit numbers (--max-seqs). Here, hit number 300 and sensitivity 5.7 are the default values used by MMseqs2.

**
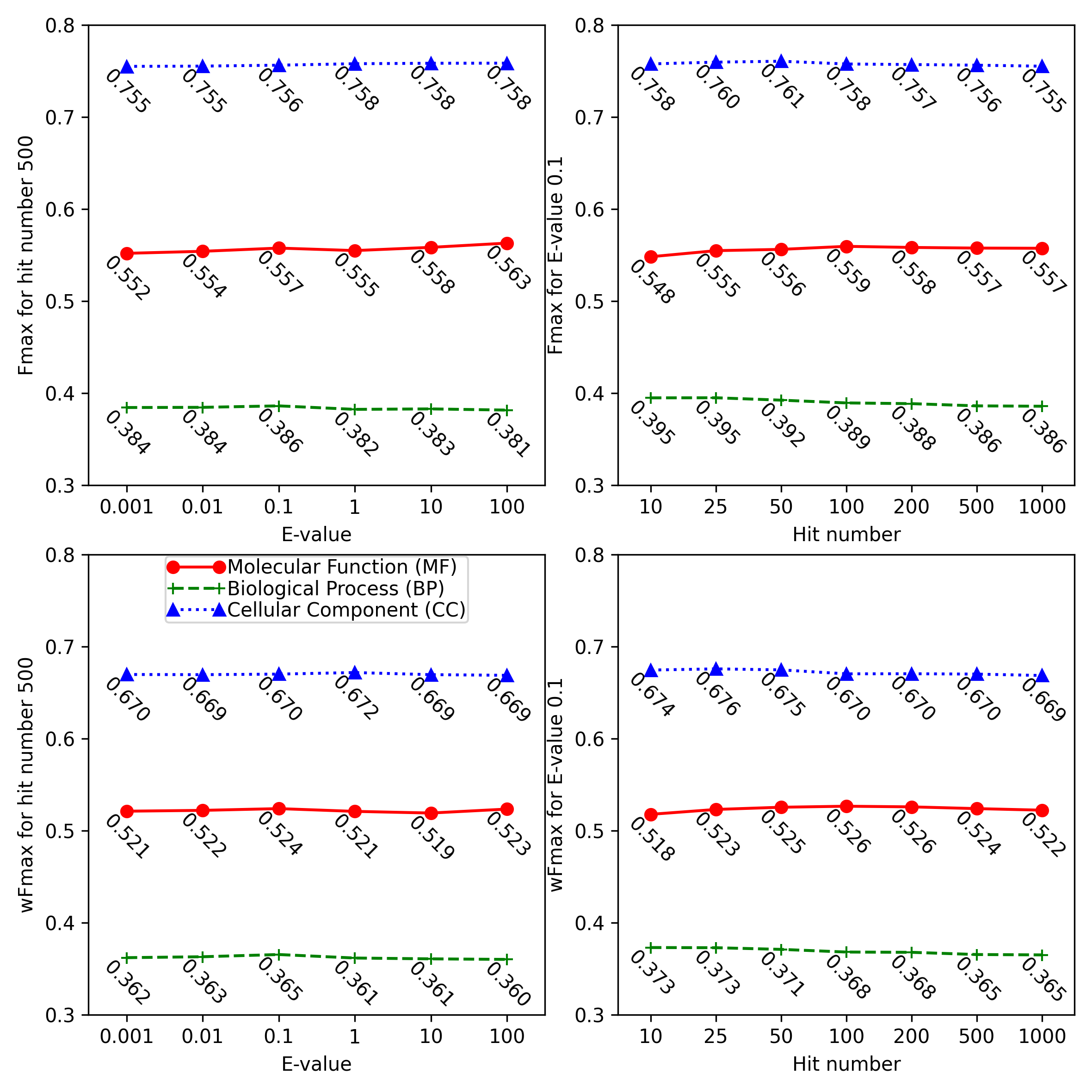
Figure S10.** The wFmax and Fmax values for GO prediction by BLASTp using different E-value cutoffs (-evalue) and hit numbers (-max_target_seqs). Here, hit number 500 is the default value used by BLASTp. E-value 0.1 is the optimal cutoff to achieve the best GO prediction accuracy.

**
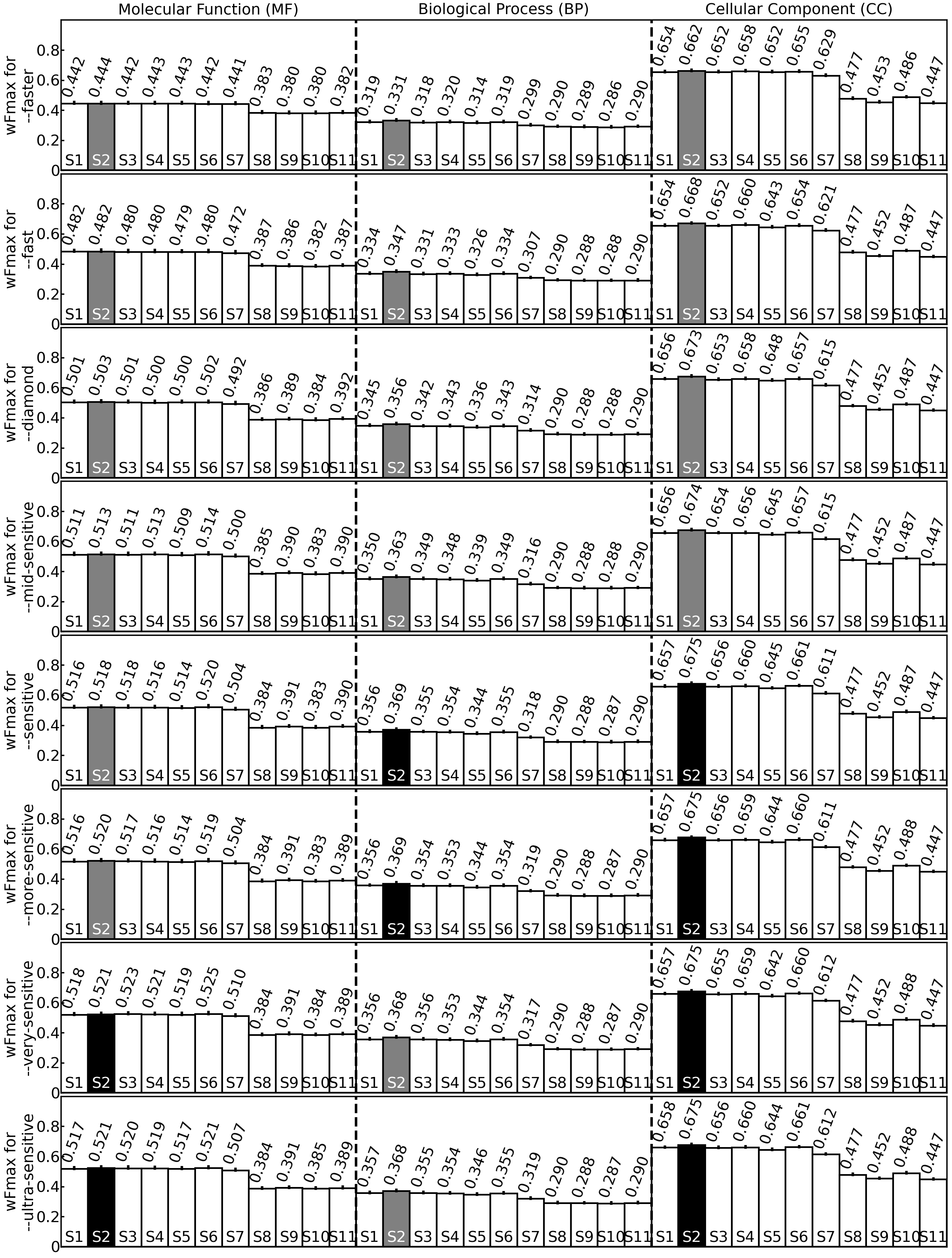
Figure S11.** The wFmax values for GO prediction by DIAMOND using 11 scoring functions (different bars in a row) and different sensitivity levels (different rows). The lengths of the error bars equal to the standard error of mean (SEM) of the per protein wFmax values. Grey bars indicate the highest wFmax value for each DIAMOND sensitivity mode. Black bars indicate the highest wFmax value for that GO aspect among all DIAMOND sensitivity modes.

**
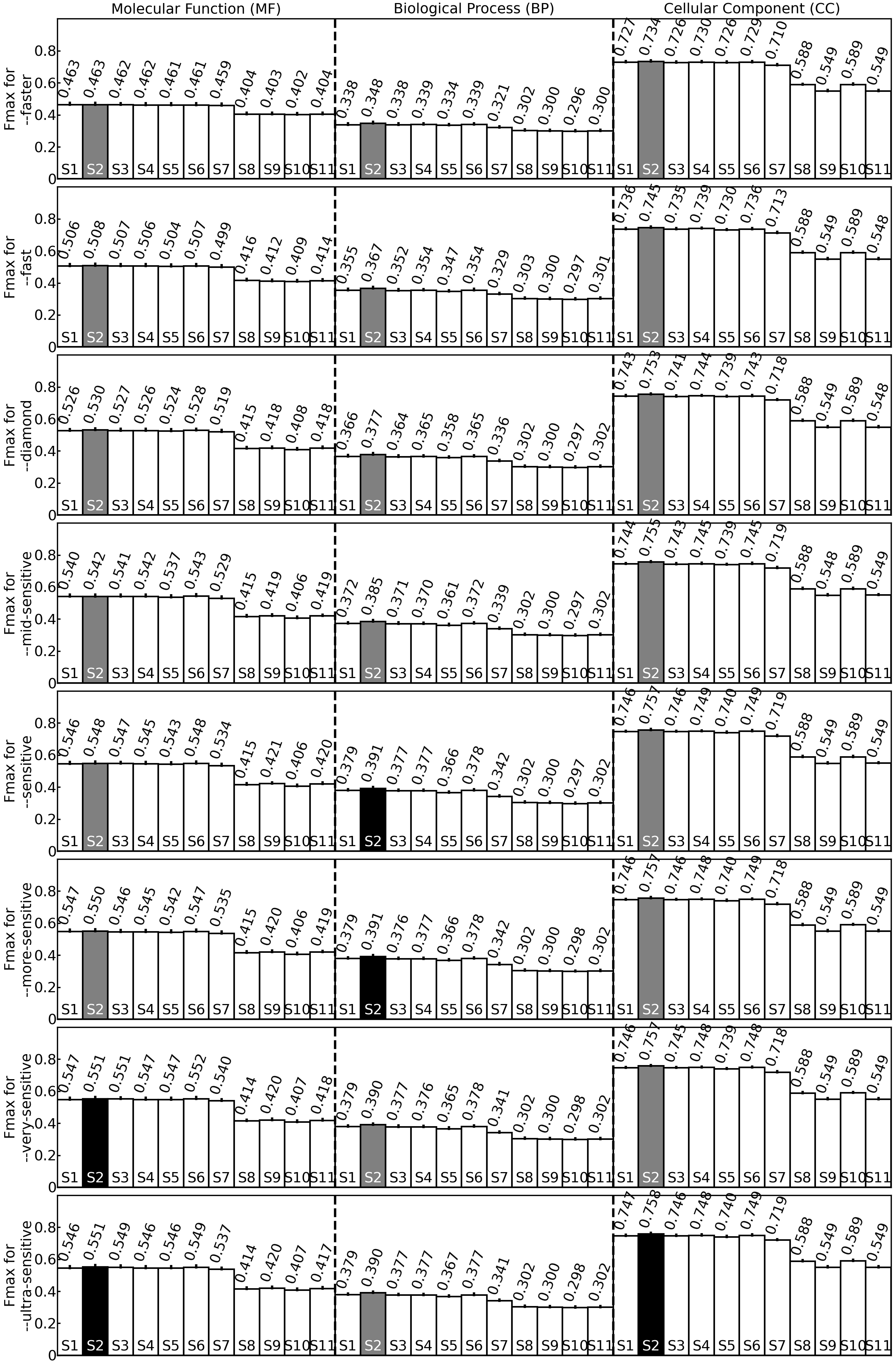
**

**Figure S12.** The Fmax values for GO prediction by DIAMOND using 11 scoring functions (different bars in a row) and different sensitivity levels (different rows). The lengths of the error bars equal to the standard error of mean (SEM) of the per protein Fmax values. Grey bars indicate the highest Fmax value for each DIAMOND sensitivity mode. Black bars indicate the highest Fmax value for that GO aspect among all DIAMOND sensitivity modes.

**
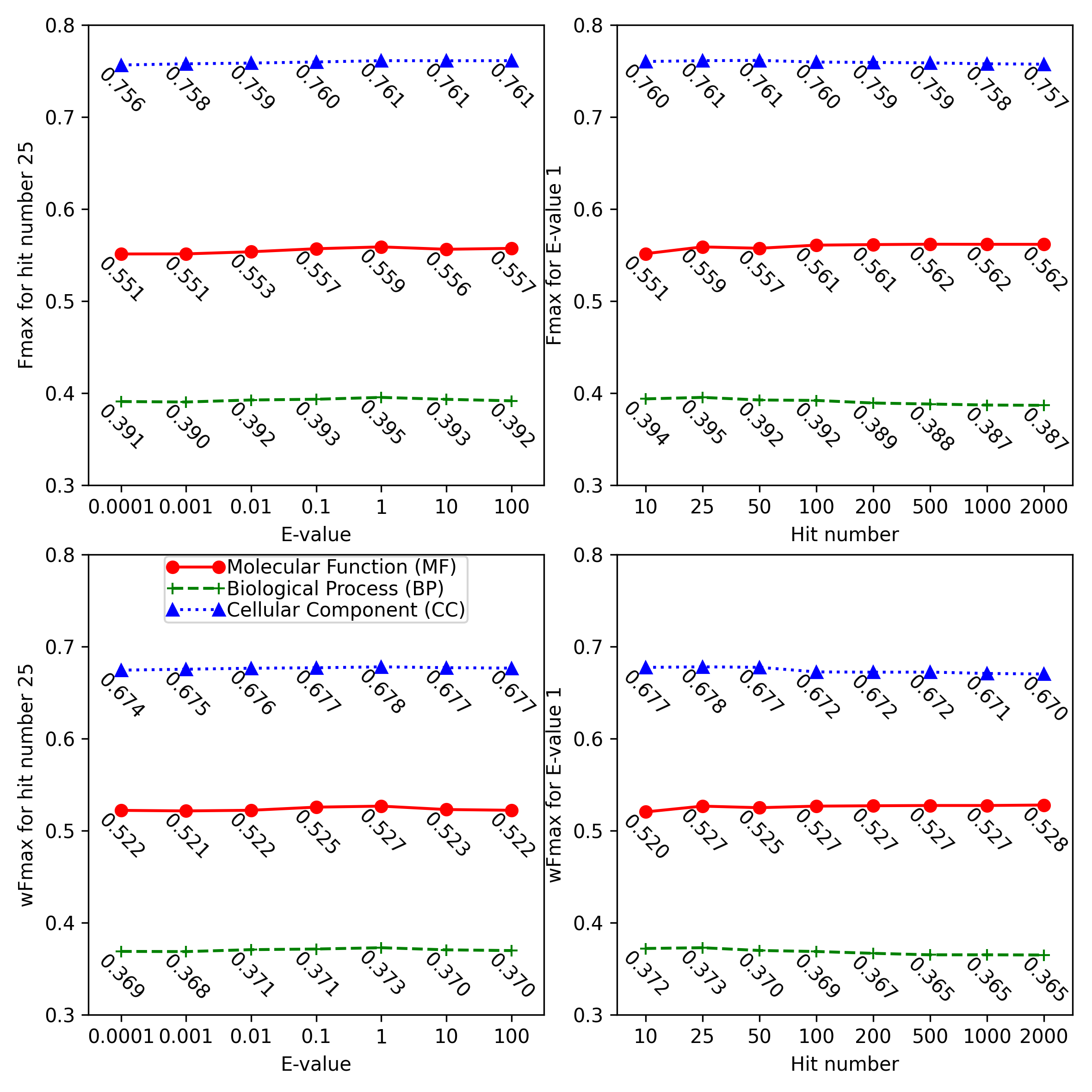
Figure S13.** The wFmax and Fmax values for GO prediction by DIAMOND using different E-value cutoffs (--evalue) and hit numbers (--max-target-seqs). Here, hit number 25 is the default value used by DIAMOND. E-value 1 is the optimal cutoff to achieve the best GO prediction accuracy.

**
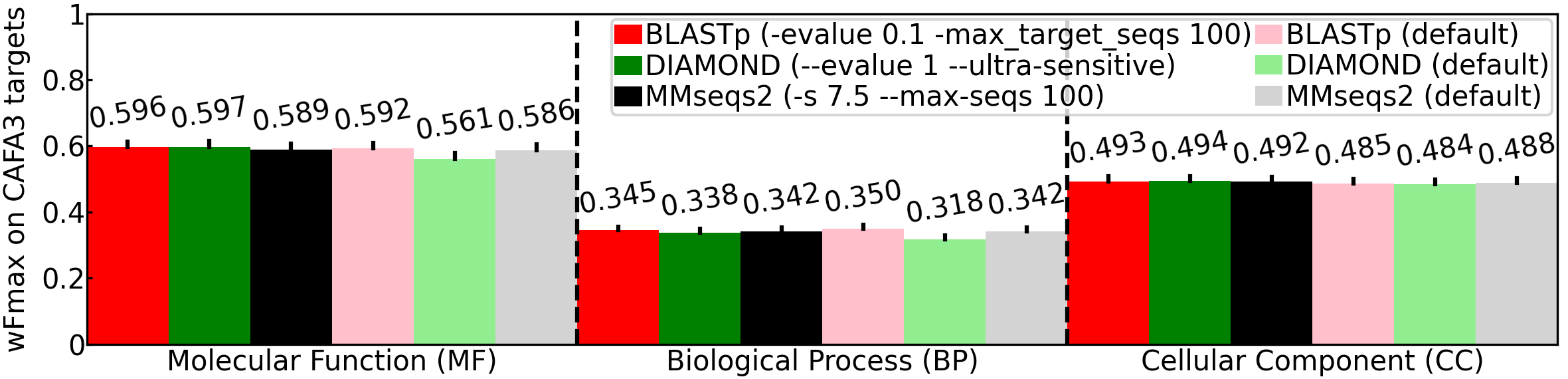
**

**Figure S14.** The wFmax values for GO prediction on the CAFA3 set using our optimized parameters (optimized on the main benchmark dataset, dark color bars) and default parameters (light color bars) for each tool. The error bar lengths reflect the standard error of mean (SEM) of weighted F-measure values per protein. The CAFA3 dataset consists of 66841 training proteins with GO annotations before September 2016, and 1222 testing proteins with new GO terms annotated on or before June 2017 but after January 2017.

**Supporting Table**

**Table S1.** Number of protein targets in different subsets of the benchmark dataset.

| Subset | MF targets | BP targets | CC targets | All targets |
| --- | --- | --- | --- | --- |
| Limited knowledge (LK) | 597 | 308 | 237 | 1024 |
| No knowledge (NK) easy | 271 | 684 | 1585 | 2160 |
| No knowledge (NK) hard | 248 | 608 | 639 | 1106 |
| All | 1119 | 1609 | 2468 | 4303 |
